# Impacts of Maternal Nutrition on Vascularity of Nutrient Transferring Tissues during Gestation and Lactation

**DOI:** 10.3390/nu7053497

**Published:** 2015-05-13

**Authors:** Kimberly A. Vonnahme, Caleb O. Lemley, Joel S. Caton, Allison M. Meyer

**Affiliations:** 1 Department of Animal Sciences, North Dakota State University, Fargo, ND 58102, USA; E-Mail: Joel.Caton@ndsu.edu; 2 Department of Animal and Dairy Sciences, Mississippi State University, Mississippi State, MS 39762, USA; E-Mail: CLemley@ads.msstate.edu; 3 Division of Animal Sciences, University of Missouri, Columbus, MO 65210, USA; E-Mail: MeyerAll@missouri.edu

**Keywords:** mammary gland, placenta, small intestine, uterine blood flow

## Abstract

As the demand for food increases with exponential growth in the world population, it is imperative that we understand how to make livestock production as efficient as possible in the face of decreasing available natural resources. Moreover, it is important that livestock are able to meet their metabolic demands and supply adequate nutrition to developing offspring both during pregnancy and lactation. Specific nutrient supplementation programs that are designed to offset deficiencies, enhance efficiency, and improve nutrient supply during pregnancy can alter tissue vascular responses, fetal growth, and postnatal offspring outcomes. This review outlines how vascularity in nutrient transferring tissues, namely the maternal gastrointestinal tract, the utero-placental tissue, and the mammary gland, respond to differing nutritional planes and other specific nutrient supplementation regimes.

## 1. Introduction

The world’s population reached 7 billion in 2012, and is projected to be greater than 9 billion by 2050 [1]. Continued economic and population growth in developing countries is expected to increase world-wide demand for meat, milk, and eggs [2]. Increased demand will require rapid development of new technologies to improve food animal production systems in the face of limited land and water, competition with bioenergy, and increasing environmental and animal welfare regulations. With this in mind, it is imperative that we enhance livestock production efficiency by optimizing the function of nutrient transferring tissues, namely the gastrointestinal tract, placenta, and mammary gland. Large animal research ensures food safety and improves the quality and affordability of meat and milk, as well as serve as biomedical models. Understanding nutrient transferring tissue efficiency is critical, not only for the growth and development of the dam, but also for the offspring. Animal models are particularly important to human health in the area of developmental programming. Maternal delivery of nutrients to the fetus can be reduced by decreased placental function, just as poor neonatal nutrient delivery is impaired by poor mammary gland development. Both the placenta and mammary gland are dependent upon the maternal gastrointestinal tract to adequately extract and deliver dietary nutrients. While it is well known that the gastrointestinal tract, placenta, and mammary gland adapt to different nutritional stimuli, how these tissues adapt to different nutritional stressors (*i.e.*, inappropriate nutrient supply, conditional increases in nutrient demand, specific nutrient imbalances, *etc.*) may be keys for enhancement of nutrient transfer efficiency.

The area of developmental programming and the study of nutrient exchange in tissues is of interest to both agriculture and human health. Obviously, research done in agriculturally relevant species such as the cow, sheep, and pig will directly relate to those species. However, these large domesticated animals are also a primary model for human research. The advantages of domesticated animal models are important for moving concepts from the laboratory to human health applications (http://adsbm.msu.edu/) [3]. In particular, sheep have been used as a relevant model for biomedical research for humans because there is a large body of data on reproductive function, including pregnancy, available [4,5,6,7,8,9,10,11,12]. A variety of animal species, from laboratory rodents to domestic ruminants and non-human primates, have been used to study pregnancy. This review will highlight how nutrient transferring tissues are affected in several domesticated animals, with more data presented from sheep. While no animal model truly recapitulates processes happening in the human, the sheep model has some resemblances with human reproduction which include: 1) a relatively long gestation length (*i.e*., ~150 days); 2) generally carries singleton or twin fetuses; and 3) the villous tree of the sheep cotyledon is structurally similar to the human placenta. Furthermore, the ewe is a robust model allowing for surgical manipulations for repeated maternal and fetal sampling/data collection to determine how both maternal and fetal tissues respond to physiological stimuli (such as nutritional manipulations). Moreover, there is a wealth of information in the sheep as it has been used extensively over the past 40 years as a model of human pregnancy [9]. Additional appreciable features of the sheep model which are relatively comparable to human fetal development include: neonatal thermoregulation, metabolic regulation, specific organ growth rate, fetal protein turnover, maturity of hypothalamic-pituitary-thyroid-adrenal axis and others, as summarized by Symonds *et al*. [6]. Furthermore, a sheep model allows for performing controlled studies using a greater number of animals, and for obtaining a large amount of tissues from the relevant physiological states.

### How Pregnancy Alters Physiology and Metabolism

During pregnancy, maternal physiologic state is associated with significant but reversible modulations to meet metabolic demand as well as alterations to the endocrine and cardiovascular systems. Endocrine secretions from the conceptus allows for communication between the maternal endometrium and fetal membranes of the placenta during mid- to late-gestation, helping to ensure proper nutrient and waste exchange during exponential growth of the fetus. Moreover, it is evident that pregnancy alters vascularity of the small intestine [13] allowing for greater uptake of nutrients. Maternal cardiovascular capacity also changes dramatically during pregnancy, with decreases in systemic arterial blood pressure and vascular resistance and increases in cardiac output, heart rate, heart stroke volume, and blood volume [14]. Mean arterial pressure decreases in early pregnancy and persists throughout gestation in several mammalian species. The decrease in arterial pressure (~5%–10% decrease) is minor compared to the approximate 20%–30% decrease in total peripheral vascular resistance. Maternal cardiac output has been shown to increase as much as 30%–40% in pregnant *vs.* non-pregnant ruminants [14]. Therefore, the increase in cardiac output is associated with a fall in systemic vascular resistance, allowing researchers to characterize pregnancy as a state of systemic vasodilation resulting in profound increases in total systemic flows to all vascular beds. In addition to the changes in the maternal cardiovascular system during pregnancy, it is also noteworthy to point out that the majority of mammals will return to the non-pregnant levels of cardiovascular function 2–5 weeks postpartum indicating that vascularity is important during involution and repair of the uterus.

Two important contributors to the alterations in the cardiovascular system include vascular endothelial growth factor (VEGF) and nitric oxide (NO). Vascular endothelial growth factor and its receptors are distributed widely throughout adult and fetal tissues [15,16,17,18,19]. While many angiogenic factors are associated with fetal development, VEGF is one of the most potent factors associated with fetal and placental vascular growth in many species including sheep [17,18,20,21,22], cow [23,24], and pig [25,26]. While changes in VEGF in the maternal circulation do not appear to change in cattle or sheep (Table 1), there is an increase in fetal circulation in swine [26]. Moreover, VEGF mRNA increases in the pig placenta, but is not altered in ruminants as gestation advances. In addition to VEGF stimulating angiogenesis, VEGF upregulates nitric oxide (NO) production by endothelial cells [27,28,29,30]. While NO metabolites increase as gestation advances in the ewe [22] and cow ([31]; Table 1), similar information has not been reported for pigs. Moreover, while endothelial nitric oxide synthase (eNOS) mRNA expression does not appear to significantly change as gestation advances, it is known that several NO mediated vascular events are occurring in the placenta [32,33,34,35] and the mesenteric arteries [36,37].

**Table 1:** Changes in VEGF protein and placental mRNA as well as nitric oxide metabolite (NOx) and eNOS mRNA from mid- to late-gestation in cattle, sheep, and swine.

	In Circulation	mRNA in Fetal Placenta	mRNA in Maternal Placenta
VEGF			
Cow	Maternal: No change ^[23]^	8% increase ^[38],^*	43% decrease ^[38],^*
Sheep	Maternal: No change ^[22]^	36% increase ^[21],^*	14% decrease ^[21],^*
Pig	Fetal: 83% increase ^[26]^	50% increase ^[26]^	18% increase ^[26]^
Nitric Oxide	NOx	eNOS	eNOS
Cow	210% increase ^[31]^	29% increase ^[38],^*	49% increase ^[38],^*
Sheep	260% increase ^[22]^	37% increase ^[21],^*	42% increase ^[21],^*
Pig	**	**	**

*Not significant at *p* ≤ 0.05; ** Unknown.

While there are many reviews that focus on how different maternal nutritional levels impact the growth and development of the offspring, this review will focus on how differing maternal nutritional levels impact nutrient transferring tissues. We hypothesize that in order to develop potential therapeutics that could spare negative influences on the offspring, functions of the small intestine, placenta and/or the mammary gland must become more efficient at nutrient delivery. Core mechanisms that regulate vascularization and blood flow within key nutrient transferring tissues are beginning to be understood, and likely are a major determinant of animal growth and nutrient utilization.

## 2. Maternal Gastrointestinal Tract during Pregnancy

The maternal gastrointestinal tract is critical for nutrient acquisition but is also a major nutrient sink during pregnancy. In ruminants, the liver and gut consume approximately 40% of maintenance energy demands [39,40]. Within the gastrointestinal tract, which consumes approximately 20% of maintenance energy [41,42], a majority of these nutrient resources are consumed by ion transport, protein turnover, enzyme secretion, and active transport [43,44]. The dam needs to maintain functional capacity of the gastrointestinal tract, even though a decrease in nutrient use by this tissue may be necessary, especially in cases of gestational nutrient restriction. Pregnancy [13,45], stage of gestation [46,47,48], and nutrient intake (Table 2) affect intestinal tissue mass and growth indices. Alternatively, some studies revealed minimal or no differences in small intestinal mass in response to pregnancy [46], stage of gestation [49], or nutrient intake (Table 3), leading us to hypothesize that changes are influenced by species, age/parity, previous nutritional plane, diet type, and other potential metabolic mechanisms.

**Table 2:** Histological measurements to assess vascularity of nutrient transferring tissues.

Measure	Calculation	Used to Assess
Capillary Area Density (**CAD**)	total capillary area ÷ tissue area	blood flow
Capillary Surface Density (**CSD**)	total vascular surface ÷ tissue area	nutrient exchange
Capillary Number Density (**CND**)	total number of vessels ÷ tissue area	vascular branching
Area Per Capillary (**APC**)	total capillary area ÷ number of vessels	capillary size
Total Vascularity	CAD × tissue mass	total vascular bed of tissue

The small intestine, which has a high degree of plasticity, is a focus of research during pregnancy because of its role in nutrient acquisition, nutrient utilization, and immunocompetence. Interestingly, the similarities in nutrient-transferring functions associated with the small intestine and the placenta have allowed many research techniques to be adapted and shared across tissues and within studies, including vascular perfusion and histological measures of vascularity (Table 2). In the small intestine, most measures have focused on the proximal jejunum, where both digestion and absorption occur. As with the placenta, these measures of vascularity have been responsive to pregnancy, stage of gestation, and nutritional status during pregnancy. Capillary area density and total vascularity of the jejunum increased in pregnant multiparous ewes, and total vascularity increased from mid- to late gestation [49]. In another study, jejunal capillary surface density and size increased from mid- to late gestation, while capillary number density decreased from early to late gestation in primiparous ewes [47]. Jejunal capillary area density and total vascularity also decreased from early to mid-gestation, then increased from mid- to late-gestation, possibly due to the maternal transition from the anabolic to catabolic state of pregnancy [47]. Additionally, capillary area density of the jejunum also increased from mid- to late-pregnancy in beef cattle [48]. These studies suggest that vascularity of the small intestine may play more of a role as the nutrient demand of fetal growth increases during late gestation. We hypothesize that we are observing increases in vasodilation, which would decrease resistance to blood flow, and allow for increased nutrient acquisition.

Overall, small intestinal size and function, including vascularity, are an active component of maternal physiological adaptations during pregnancy. These adaptations occur in response to increasing metabolic demands of advancing gestation, altered nutritional plane, and inappropriate levels of specific nutrients (Table 2). This should come as no surprise given that the small intestine is responsive to changes in intake and metabolic demand [50].

### Vascularization of the Maternal Small Intestine in Response to Different Nutritional Treatments

Effects of nutrient intake in the face of pregnancy demands in ruminants (especially sheep) have been studied extensively by our group, and vascular changes due to nutrient intake, including nutrient restriction, overnutrition, and supranutritional selenium diets, are summarized in Table 2. Jejunal vascularity is not affected by gestational nutrition in beef cows [48], but it has been variable in sheep. Vascularity measures have increased, decreased, or not been affected by nutrient restriction during pregnancy in sheep, even though the mass of the small intestine is generally decreased by nutrient restriction (Table 2). Interestingly, mature ewes undergoing nutrient restriction have increased jejunal capillary area density as the mass of their small intestine decreases, allowing for similar total vascularity of the small intestine in restricted compared with adequately nourished ewes [49]. This phenomenon is not observed in ewe lambs (~8.5 month age at breeding) which demonstrated both decreased capillary area density and small intestinal mass, resulting in a decrease of total vascularity [51]. Because young females are still growing, pregnancy may slow intestinal growth and affect development, resulting in decreased mass and angiogenesis during times of combined physiological and nutritional stress. More studies need to be conducted in order to determine how age, parity, and stage of pregnancy contribute to vascular changes in the small intestine.

Overfeeding ewes during gestation affects vascularity of the small intestine less often than nutrient restriction, despite an increase in mass of the small intestine (Table 2). The increased mass associated with increased feed intake may allow for enough of an increase in overall vascularity [52] that increased vascularity per unit of tissue is not necessary in these instances to meet increased nutrient demand associated with pregnancy.

Factors affecting regulatory mechanisms of vascularity of the small intestine are not well understood. Nutrient intake appears to be a major factor, as growing steers with greater feed intake have increased capillary area, number, and surface densities as well as total vascularity [53]. This suggests that vascularity of the small intestine may follow nutrient intake to allow for greater nutrient absorption, which seems logical. In general, our studies with sheep support this notion, although fetal and placental signals likely complicate this relationship during pregnancy. The VEGF and NO systems play a role in modulating changes in vascularity and they are affected by nutritional plane during gestation (Table 4). In general, expression of VEGF and its receptors within intestinal tissues are altered more than eNOS or soluble guanylate cyclase (GUCY1B3). Additionally, expression of VEGF receptors are down regulated by supranutritional levels of selenium during pregnancy [52,54], whereas even within supranutritional selenium, VEGF is increased with dietary selenium (15 ppm > 3 ppm; 50). *In vitro* systems have demonstrated that VEGF delivery to the small intestine increases vascularity [55]. In our studies, vascularity of the small intestine and expression of genes for angiogenic factors change coordinately [52]. or, at times, in opposite directions [51,52,54]. Additionally, gene expression is not always altered at the same time as a change in vascularity, and *vice versa* (Table 3 and Table 4). This could be due to the time of sampling of tissues, or differences in mRNA and protein expression.

The small intestine exhibits a high degree of plasticity and responds to nutrient presence in the lumen, immunomodulatory factors, hormones, growth factors, local cell communication, and microbial and host interactions [56,57,58,59]. Thus, changes in the vascularity and size of the small intestine in pregnant animals could be due to increased intake (which is typical when food is consumed ad libitum in most species), increased nutrient demand and resulting hormone and growth factor signals, and signals coming directly from the placenta or fetus. For example, estradiol-17β implants alter jejunal capillary number density and crypt cell proliferation and interact with dietary linseed meal (contains diphenolic compounds with estrogenic-like activity) and results in changes in impact mRNA expression of VEGF receptors and GUCY1B3 in ovariectomized ewes [60]. Additionally, blood flow to the small intestine increases with estradiol-17β infusion [14]. Thus, estradiol during pregnancy may be one of the contributing factors in adaptation of the intestine to pregnancy.

Proper and efficient function of the small intestine is necessary for the dam to acquire and deliver nutrients to the fetus during pregnancy. In all of our sheep studies, nutrient restriction in mid- and late-gestation reduces fetal growth (Table 3). The overall decrease in the nutrient transferring capacity of the small intestine due to changes in mass, vascularity, or other factors likely plays a role in decreased fetal growth. Although the maternal small intestine appears to increase vascularity during nutrient restriction in some studies [49], this has not occurred in all experiments. It is possible that successful maternal adaptation of the gut during nutrient restriction is able to prevent a decrease in nutrient delivery to the fetus in some animals, but more research is necessary. While the maternal gastrointestinal tract has the first opportunity to utilize nutrients after consumption, most data from stable isotope studies indicate [61]. that the gut is a highly competitive tissue, deriving the majority of its amino acids from arterial compared with luminal supplies. Maternal nutrient sourcing (arterial or luminal) has not been well studied during advancing pregnancy. If additional nutrients are used by the small intestine due to poor adaptation to nutrient restriction or pregnancy, decreased nutrients would be available for delivery to the fetus. Additionally, if the gastrointestinal tract is increasing in mass due to overnutrition during gestation (Table 3), this may also divert nutrients from fetal growth.

**Table 3:** Vascularity and mass of the maternal small intestine in ovine and bovine models for investigating the interrelationships of nutrition and reproduction.

Treatment	Stage	Impact on Vascularity *	Impact on Actual Mass	Impact on Production
**Cattle—Gestation**				
Control (CON) *vs.* Restricted (RES) Day 30 to 125 gestation ^[48]^	Mid gestation (Day 125)	NS ^1^	NS	Fetal weight: NS
Control (CON) *vs.* Restricted (RES) and realimented RES: Day 30 to 125, Realimented: Day 125 to 245 gestation ^[48]^	Late gestation (Day 245)	NS	NS	Fetal weight: NS
**Sheep—Gestation**				
Control (CON) *vs.* Restricted (RES) Day 50 to 90 gestation ^[45,49]^	Mid gestation (Day 90)	CAD: RES greater than CON	CON > RES	Fetal weight: NS
Control (CON) *vs.* Restricted (RES) Day 50 to 130 gestation ^[45,49]^	Late gestation (Day 130)	CAD: RES greater than CON	CON > RES	Fetal weight: CON greater than RES
Control (CON) *vs.* Restricted (RES) Day 64 to 135 gestation ^[51]^	Late gestation (Day 135)	CAD 17% less, APC 16% less, and total vascularity 35% less in RES *vs.* CON	20% less in RES *vs.* CON	Fetal weight 10% less in RES *vs.* CON
Control (CON) *vs.* RES-CON ^2^ Day 50 to 132 of gestation ^[62]^	Late gestation (Day 132)	NS	NS	Fetal weight: NS
Control (CON) *vs.* CON-RES ^2^ Day 50 to 132 of gestation [62]	Late gestation (Day 132)	NS	17% less in CON-RES *vs.* CON	Fetal weight 13% less in CON-RES *vs.* CON
Control (CON) *vs.* RES-RES ^2^ Day 50 to 132 of gestation ^[62]^	Late gestation (Day 132)	NS	13% less in RES-RES *vs.* CON	Fetal weight 14% less in RES-RES *vs.* CON
Control (CON) *vs.* Restricted (RES) Day 40 gestation to parturition ^[52,63]^	Within 24 h post-partum	CSD 19% less in RES *vs.* CON	NS	Birth weight 9% less in RES *vs.* CON
Control (CON) *vs.* Overnourished (HIGH) Day 0 to 50 gestation ^[47]^	Early gestation (Day 50)	NS	19% greater in HIGH *vs.* CON	Fetal weight: NS
Control (CON) *vs.* Overnourished (HIGH) Day 0 to 90 gestation ^[47]^	Mid gestation (Day 90)	NS	43% greater in HIGH *vs.* CON	Fetal weight: NS
Control (CON) *vs.* Overnourished (HIGH) Day 0 to 130 gestation ^[47]^	Late gestation (Day 130)	NS	NS	Fetal weight 11% less in HIGH *vs.* CON
Sheep—Gestation				
Control (CON) *vs.* Overnourished (HIGH) Day 40 gestation to parturition ^[52,63]^	Within 24 h post-partum	Total vascularity 38% greater in HIGH *vs.* CON	28% greater in HIGH *vs.* CON	Birth weight: NS
Adequate Se (ASe; 0.3 ppm) vs. High Se (HSe; 3 ppm) Day 0 to 135 gestation ^[51]^	Late gestation (Day 135)	NS	10% greater in HSe *vs.* ASe	Fetal weight 10% greater in HSe *vs.* ASe
Adequate Se (ASe; 0.3 ppm) *vs.* High Se (HSe; 3 ppm) Day 0 to 132 gestation ^[62]^	Late gestation (Day 132)	NS	NS	Fetal weight: NS
Adequate Se (ASe; 0.3 ppm) *vs.* High Se (HSe; 3 ppm) Day 0 gestation to parturition ^[52,63]^	Within 24 h post-partum	NS	17% less in ASe *vs.* HSe	Birth weight: NS
Adequate Se (ASe; 0.14 ppm) *vs.* High Se (HSe; 3 or 15 ppm) Day 50 to 134 gestation ^[64]^	Late gestation (Day 134)	APC 33% less in HSe *vs.* ASe	NS	Fetal weight: NS
High-Se wheat *vs.* Sodium selenate (3 ppm) Day 50 to 134 gestation ^[64]^	Late gestation (Day 134)	CND 25% greater and CSD 73% greater in selenate *vs.* wheat	NS	Fetal weight: NS
3 ppm *vs.* 15 ppm of Se (both sodium selenate) Day 50 to 134 gestation ^[64]^	Late gestation (Day 134)	NS	NS	Fetal weight: NS
**Sheep—Lactation**				
Control (CON) *vs.* Restricted (RES) Day 40 gestation to parturition ^3^ ^[52,65]^	Early lactation (21 day)	CSD 5% greater in CON *vs.* RES	NS (both increased post-partum)	Milk production 22% less in RES *vs.* CON
Control (CON) *vs.* Overnourished (HIGH) Day 40 gestation to parturition^3^ ^[52,65]^	Early lactation (21 day)	Total vascularity 13% greater in HIGH *vs.* CON	NS (both increased post-partum)	Milk production: NS
Adequate Se (ASe; 0.3 ppm) *vs.* High Se (HSe; 3 ppm) Day 0 gestation to parturition^3^ ^[52,65]^	Early lactation (21 day)	APC 23% greater in HSe *vs.* ASe	NS (both increased post-partum)	Milk production 10% greater for HSe *vs.* ASe

* Vascularity measurements are included in Table 3. CAD = capillary area density; CSD = capillary surface density; CND = capillary number density; APC = area per capillary; ^1^ NS = not significant; ^2^ RES-CON = RES from Day 50 to 90 of gestation, CON from Day 90 to 132 of gestation; CON-RES = CON from Day 50 to 90 of gestation, RES from Day 90 to 132 of gestation; RES-RES = RES from Day 50 to 132 of gestation; ^3^ Fed to meet NRC requirements for lactation with adequate Se post-partum.

**Table 4:** Expression of VEGF and NO system mRNAs in the small intestine of ewes.

Treatment	Stage	Gene Expression ^2^
**Sheep—Gestation**		
Control (CON) *vs.* Restricted (RES) Day 64 to 135 gestation ^[51,54]^	Late gestation (Day 135)	VEGF, FLT1, KDR: RES > CON
Control (CON) *vs.* Restricted (RES) Day 40 gestation to parturition ^[52,63]^	Within 24 h post-partum	FLT1: RES > CON
Control (CON) *vs.* Overnourished (HIGH) Day 40 gestation to parturition ^[52,63]^	Within 24 h post-partum	VEGF, FLT1, and NOS3: HIGH > CON
Adequate Se (ASe; 0.3 ppm) *vs.* High Se (HSe; 3 ppm) Day 0 to 135 gestation ^[51,54]^	Late gestation (Day 135)	NS ^3^
Adequate Se (ASe; 0.3 ppm) *vs.* High Se (HSe; 3 ppm) Day 0 gestation to parturition ^[52,63]^	Within 24 h post-partum	FLT1: ASe > HSe
Adequate Se (ASe; 0.14 ppm) *vs.* High Se (HSe; 3 or 15 ppm) Day 50 to 134 gestation ^[54,64]^	Late gestation (Day 134)	^4^ KDR: ASe > HSe
High-Se wheat *vs.* Sodium selenate (3 ppm) Day 50 to 134 gestation ^[54,64]^	Late gestation (Day 134)	^4^ NS
3 ppm *vs.* 15 ppm of Se (both sodium selenate) Day 50 to 134 gestation ^[54,64]^	Late gestation (Day 134)	^4^ VEGF: 15 ppm > 3 ppm
**Sheep—Lactation**		
Control (CON) *vs.* Restricted (RES) Day 40 gestation to parturition ^[52,64,65]^	Early lactation (21 day)	NS
Control (CON) *vs.* Overnourished (HIGH) Day 40 gestation to parturition ^[52,64,65]^	Early lactation (21 day)	NS
Adequate Se (ASe; 0.3 ppm) *vs.* High Se (HSe; 3 ppm) Day 0 gestation to parturition ^1^	Early lactation (21 day)	NS

^1^ Fed to meet NRC requirements for lactation with adequate Se post-partum; ^2^ FLT1 = fms-related tyrosine kinase 1 (VEGF receptor 1), KDR = kinase insert domain receptor (VEGF receptor 2), GUCY1B3 = soluble guanylate cyclase (NO receptor); ^3^ NS = not significant; ^4^ NOS3 and GUCY1B3 were not measured.

## 3. Placenta

Unlike most eutherians, livestock have non-invasive placentas. Gross morphology of the ruminant placenta is termed cotyledonary, and pigs and horses have a diffuse type of placentation. Microscopically, livestock species have epitheliochorial placentation, with six cellular layers separating maternal and fetal blood. Some argue that ruminant placentas are better classified as syndesmochorial due to their formation of giant trophoblast cells by chorionic and uterine epithelia [66,67,68]. In swine, the diffuse placenta has chorionic villi attachment distributed over the entire surface of the chorion. The presence of primary and secondary rugae increases the relative surface area of attachment between the endometrium and the fetal membranes [69]. Within the large white breeds of domestic pigs, placental area of attachment continues to increase as gestation advances [70,71] and vascular development of the placenta, as measured by the density of larger blood vessels (*i.e.*, arterioles), increases ~200% in the fetal portion of the placenta [71] with maternal vascular density remaining similar [26,71] from mid- to late-gestation. In ruminants, the fetal placenta attaches to discrete sites on the uterine wall called caruncles, which are aglandular sites appearing as knobs along the uterine luminal surface of non-pregnant animals [72]. The placental membranes attach at these sites via chorionic villi in areas termed cotyledons. The caruncular-cotyledonary unit is called a placentome and is the primary functional area of physiological exchanges between mother and fetus. In the ewe, the growth of the cotyledonary mass is exponential during the first 70–80 days of pregnancy, thereafter slowing markedly until term [73]. In contrast, the placental growth in the cow progressively increases throughout gestation [24,74]. Perhaps, these alterations in growth patterns in the sheep and cow placenta help explain the change of capillary area density (*i.e.*, a blood flow related measure; [21]) that exist from mid- to late-gestation [75]. While sheep placentas remain relatively similar in weight from mid- to late-gestation, there caruncular and cotyledonary capillary area density increase ~200 and 400%, respectively [21,75]. Bovine placentas exhibit relatively modest changes in capillary area density (compared to sheep) from mid- to late-gestation with capillary area density in caruncular tissue decreasing ~30% and cotyledonary tissue increasing ~190%, with caruncular and cotyledonary tissue weights increasing ~530% and ~650%, respectively [24].

### 3.1. Vascularity of the Placenta and Utero-Placental Blood Flow in Response to Different Nutritional Treatments

Several authors have established that many of the models of placental insufficiency are, in part, due to reduced placental vascularity and uterine or umbilical blood flows (reviewed in [76,77,78]). When placental growth is restricted in ewes, umbilical and uterine blood flows are reduced, limiting fetal growth [79]. Recently, in our laboratories, we have investigated if placental vascularity and uterine/umbilical blood flows are impacted by different maternal dietary treatments. In sheep, while we have observed reductions in umbilical blood flow [80] and increases in arterial indices of resistance [81], we have not observed alterations in placental capillary densities [82,83]. After 30 days whereby a 40% reduction in intake was applied (*i.e.*, on Day 50), umbilical blood flow of singleton fetuses was reduced compared to adequately fed control ewes [80]. This reduction remains through late gestation (Day 130). In beef cattle, we initially hypothesized that, similar to sheep, reductions in intake would lead to reductions in uterine blood flow. In contrast, we observed that during a 110 day nutrient restriction (*i.e.*, 40% of the control diet), uterine blood flows were similar [84]. Interestingly, upon realimentation, blood flow to the ipsilateral horn increases [84]. While our work has been done with global nutrient restriction and realimentation, Perry *et al.* [85] reported that protein restriction during the first trimester of pregnancy followed by increased protein concentration during the second trimester enhances placental development and fetal growth. Increased dry matter intake has been linked to enhanced maternal insulin-like growth factor-1 during late pregnancy [86]. If exogenous insulin-like growth factor-1 is administered to the dam, there is increased glucose and amino acid uptake by both fetal and maternal tissues [87]. Uteroplacental blood flow is undoubtedly associated with fetal growth and development; however, specific nutrient transport across the feto-placental unit rely not only on adequate blood flow but also on adequate nutrient transporter densities.

For our beef cattle studies, color Doppler ultrasonography was utilized. Prior to our studies, others have used this non-invasive technique which has been used to measure uterine blood flow and arterial indices of resistance in cattle [88,89,90]. Foundational uterine blood flow work in beef cattle has been conducted utilizing more invasive techniques such as electromagnetic blood flow transducers [91] or infusion of deuterium water [92]. By using color Doppler ultrasonography to assess uterine blood flow and vascular resistance throughout gestation in pregnant beef cows, we were able to examine the same animal continuously throughout gestation with no surgical preparation and with minimal interference to dam. Similarly, Bollwein *et al.* [88] measured uterine blood flow in cows during the estrous cycle. They suggested that Doppler ultrasonography was a reliable method to determine uterine blood flow and did not require use of blood flow probes and/or chronically catheterized animals. Moreover, findings from this study and others suggest that the use of Doppler ultrasonography as the technique to investigate uterine blood flow during pregnancy may also constitute a reliable method. In several breeds of cattle, when Doppler ultrasonography assessed uterine hemodynamics throughout gestation (*i.e.*, Day 30–270) resistance index decreased and uterine blood flow increased exponentially with increased blood flow in ipsilateral *vs.* contralateral horns [89,93]. Moreover, resistance index was negatively correlated to uterine blood flow [93].

The hypothesis that during nutrient restriction total uterine blood flow would be reduced was rejected. Moreover, while total uterine blood flow was similar after realimentation, ipsilateral uterine blood flow was enhanced in cows that were previously restricted. In many sheep models investigated to date [76,80], nutrient restriction results in reduced uterine and/or umbilical blood flow. This could be innate species differences, or also due to parity or age of the dam. Regardless, until more beef cattle work is performed to confirm our results, caution should be used when comparing data acquired in sheep as it may not be directly applicable to beef cattle. Inanition in swine during mid-pregnancy (Day 50–90; gestation length = 114 days) resulted in no change to uterine blood flow, similar to our findings in the beef cow, and resulted in no change in weight of the total uterine mass [94]. Despite this lack of change in uterine blood flow, Hard and Anderson [94] further demonstrated that blood volume was reduced during inanition, but increased by 24% within 20 days of realimentation. Unfortunately, how realimentation influenced uterine blood flow was not measured in the Hard and Anderson study. In women experiencing hyperemesis gravidarum, uterine blood flow per 100 g of fetus is increased compared to control women [95], but the authors were unable to locate information on how normal intakes may have impacted uterine blood flow in those pregnancies. While there is a paucity of information on how restriction and/or realimentation impacts uterine blood flow, it appears most pregnant females alter their body reserves to allow continuation of adequate blood flow for the developing conceptus. However, to our knowledge, uterine blood flow in models of nutrient restriction has not been measured upon realimentation in any species.

### 3.2. Utero-Placental Amino Acids

Uteroplacental and fetal uptake of amino acids can be primarily divided into two areas of interest focusing on either metabolic pathways or transport systems [77,96,97]. From a metabolic standpoint, research has shown consistent uterine and fetal uptakes of essential amino acids [98]. However, there is evidence that amino acid derivatives, such as creatine, which is actively transported across the placenta, may provide beneficial impacts to the fetus [99]. Focusing on essential amino acids, previous research has shown net uteroplacental consumption of isoleucine, leucine, and valine, while methionine was the only essential Amino Acid (AA) showing a net uteroplacental release [98]. The uteroplacental consumption of branched-chain amino acids to their respective keto acids contributes extensively to placental glutamate production via α-ketoglutarate. This relationship allows for the establishment of a feto-placental glutamate–glutamine shuttle, whereby the fetus is dependent on this exogenous supply of glutamine from the placenta [100,101,102], which could be altered by placental insufficiency [103,104]). The ovine placenta expresses glutamine synthetase, which catalyzes the condensation of glutamate and ammonia into glutamine [98].

Branched-chain amino acids regulate mammalian target of rapamycin (mTOR), which has been implicated as a nutritional sensor, that regulates cell growth and protein synthesis via increased rates of mRNA translation through the phosphorylation of eukaryotic initiation factor 4E-binding protein 1 (4E-BP1) and the ribosomal protein S6 kinase 1 [105,106,107]. Moreover, supplementation of branched-chain amino acids to dams on a protein deficient diet can restore fetal growth and minimize the decreases in fetal organ mass and carcass fat, which is associated with increased mTOR signaling in the fetus [108]. Recent evidence in human placental cell cultures as well as choriocarcinoma cell lines indicate that amino acid transporter expression and/or activity may be regulated by mTOR signaling pathways [109]. Specifically, treatment of the BJAB cell line with rapamycin (an inhibitor of mTOR complex 1) decreases mRNA expression of multiple amino acid transporters [110]. In human primary trophoblast cell cultures, inhibition of mTOR by rapamycin reduces the activity of system A, system L, and taurine amino acid transporters; however, mTOR inhibition does not change amino acid protein expression. Therefore, amino acid transporter activation is independent of increased protein synthesis.

Multiple animal models of intrauterine growth restriction have been used to examine amino acid concentrations and flux across the uteroplacenta. Table 5 summarizes maternal and offspring measurements of branched-chain amino acids following nutrient or protein restriction in various animal models. Using a mid- to late-gestation ovine model of intrauterine growth restriction, we examined branched-chain amino acid exchange via the uteroplacenta [111]. For this experiment, singleton pregnant ewes were provided 100% (control) or 60% (restricted) of nutrient recommendations from Day 50–130 of gestation. On Day 130 of gestation, ewes were anesthetized and concurrent blood samples were collected from a catheterized maternal saphenous artery and the gravid uterine vein. Immediately following this collection, the gravid uterine horn was dissected and concurrent blood samples were collected from the umbilical vein and umbilical artery. This concurrent blood sampling procedure was used to calculate gravid uterine flux of nutrients, which is equal to uterine blood flow multiplied by the maternal arterial-venous concentration difference (maternal saphenous artery–gravid uterine vein) of any given substance. Fetal flux of nutrients was calculated by taking umbilical blood flow multiplied by the fetal venous-arterial concentration difference (umbilical vein-umbilical artery) of any given substance [111]. In this experimental model, we observed a decrease in both umbilical artery and uterine artery blood flow (Table 5). Uterine and fetal uptakes of isoleucine were decreased by nutrient restriction. Interestingly, similar uterine uptakes of leucine and valine were observed between dietary treatments; however, fetal uptake of both leucine and valine were decreased in nutrient restricted *vs.* control fed ewes.

**Table 5:** Maternal and offspring measurements of branched-chain amino acid (BCAA).

Dietary Treatment	Dependent Variable	Response
**Ovine model of nutrient restriction from Day 50 to 130** ^[80,111]^	Fetal weight (Day 130)	Decreased 15% to 20%
	Placental weight (Day 130)	Similar
	Umbilical blood flow (Day 50–110)	Decreased 20%
	**Uterine blood flow (Day 130)**	Decreased 20%
	Uteroplacental nutrient flux (Day 130)	
	Uterine uptake of isoleucine	Decreased 42%
	Fetal uptake of isoleucine	Decreased 37%
	Uterine uptake of leucine	Similar
	Fetal uptake of leucine	Decreased 60%
	Uterine uptake of valine	Similar
	Fetal uptake of valine	Decreased 69%
**Ovine model of nutrient restriction from Day 28 to 135** ^[112]^	Fetal weight (Day 135)	Decreased 15%
	**Maternal and fetal plasma BCAA**	
	Maternal concentration of isoleucine	Decreased 19%
	Fetal concentration of isoleucine	Decreased 28%
	Maternal concentration of leucine	Decreased 41%
	Fetal concentration of leucine	Decreased 38%
	Maternal concentration of valine	Decreased 43%
	Fetal concentration of valine	Decreased 35%
**Ovine model of metabolizable protein restriction from Day 100 to 130** ^[113]^	Fetal weight (Day 130)	Decreased 20% to 45%
	Placental weight (Day 130)	Similar
	Umbilical blood flow (Day 50–110)	Similar
	**Uterine blood flow (Day 130)**	Increased 50% to 80% *NS
	Maternal and Fetal BCAA (Day 130)	
	Maternal concentration of leucine	Similar
	Fetal concentration of leucine	Decreased 40% to 50%
	Maternal concentration of valine	Similar
	Fetal concentration of valine	Decreased 30% to 50%
**Porcine model of protein restriction from mating to Day 60** ^[114]^	**Maternal artery and umbilical vein BCAA**	
	Maternal artery concentration of isoleucine	Similar
	Umbilical concentration of isoleucine	Decreased 19%
	Maternal artery concentration of leucine	Similar
	Umbilical concentration of leucine	Decreased 29%
	Maternal artery concentration of valine	Similar
	Umbilical concentration of valine	Decreased 20%
**Porcine model of isocaloric diets with high or low protein compared to standard diet** ^[115]^	Litter weights; litter sizes	Similar
	**Maternal and umbilical/fetal BCAA**	
	Maternal venous concentration of isoleucine	Decreased 32%
	Umbilical/fetal concentrations of isoleucine	Similar
	Maternal venous concentration of valine	Decreased 73%
	Umbilical/fetal concentrations of valine	Similar
	Maternal venous concentration of leucine	Decreased 21%
	Umbilical/fetal concentrations of leucine	Similar
^6^ **Rodent model of protein restriction from Day 1 to term** ^[108]^	Newborn pup weights	Decreased 9% to 18%
	**Maternal and newborn BCAA**	
	Maternal concentration of isoleucine	Decreased 36%
	Newborn concentration of isoleucine	Decreased 59%
	Maternal concentration of leucine	Decreased 43%
	Newborn concentration of leucine	Decreased 60%
	Maternal concentration of valine	Decreased 52%
	Newborn concentration of valine	Decreased 68%

* NS = not statistically different at *p* < 0.05.

Other ovine models of nutrient or protein restriction have shown comparable decreases in fetal weight and branched-chain amino acid profiles (Table 5). For example, Kwon *et al.* [112] observed similar decreases in maternal and fetal concentrations of isoleucine, leucine, and valine following maternal nutrient restriction from Day 28–135 of pregnancy. Using an ovine metabolizable protein restriction model where samples were simultaneously collected, Lekatz and Vonnahme [113] showed a decrease in fetal concentrations of leucine and valine; however, maternal concentrations of leucine and valine were not different between dietary treatments. Similar to the ovine metabolizable protein restriction model, Wu *et al.* [114] observed decreased fetal concentrations of branched-chain amino acids with no difference in maternal concentrations of branched-chain amino acids following protein restriction from early to mid-pregnancy in pigs. Metzler-Zebeli *et al.* [115] observed maternal venous changes, but no differences in umbilical or fetal blood of branch-chained amino acids (Table 5). Pregnant rats which were protein restricted from Day 1 to term had a decrease (36%–68%) in both maternal and newborn concentrations of branched-chain amino acids [108]. In addition to these observations, this study also identified a critical role for branched-chain amino acid supplementation in partially rescuing fetal growth restriction induced by maternal dietary protein restriction.

In conclusion, we have made interesting observations of branched chain amino acids in several sheep models of nutrient intake; however, more research is needed to determine how the placenta adapts to other maternal stressors that impact nutrient delivery, including amino acids, fatty acids, and sugars.

## 4. How Pregnancy Prepares the Dam for Lactation

The growth and development of the mammary gland from fetal life through involution in livestock has been reviewed recently and sets the stage for the discussion below [116]. As mentioned above, dramatic changes occur within the maternal cardiovascular system during pregnancy [14] and maternal energy consumption and metabolism also are altered [117] to nourish the growing conceptus and to prepare the mammary gland for lactation. Although, lactating high producing non-pregnant dairy cattle will show a substantial increase in cardiac output compared to their non-pregnant non-lactating counterparts [118,119], this redistribution of blood flow during the transition period from the uteroplacental vasculature towards the mammary gland is still a phenomenal physiological feat to allow for peak lactation shortly after parturition.

Mammary gland growth, milk yield, and mammary tissue DNA content was influenced by energy and protein intake in sows during lactation [120]. Moreover, increased dietary lipids in peripubertal ewe lambs results in increased mammogenesis [121]. While we have observed no effects of nutrition on mammary gland growth in our beef cow models [122], we do observe differences in ewes. Development of the mammary gland is unique to ewes and unlike other livestock, sheep do not exhibit post-parturient growth of the mammary gland [123]. Our laboratory has recently reported that maternal selenium supplementation as well as differing levels of maternal nutrition during mid- to late-gestation alters colostrum yield and mammary gland microanatomy [65,124,125,126]. Mammary gland alveolar epithelium has an increased proliferation index in overnourished ewes compared with restricted and adequately fed ewes indicating that the increased level of nutrients stimulated alveolar growth [124]. This increase in alveolar proliferation may be indicative of an earlier differentiation of the gland, due to overnourished ewes exhibiting decreased estradiol-17β and progesterone concentrations in circulation compared to restricted and adequately fed ewes [86,127]. Decreases in estradiol-17β reduce glucocorticoid-binding protein, allowing free cortisol to further complete cellular differentiation in preparation for lactogenesis [128]. Overnourished ewes have elevated levels of cortisol [86,127], potentially allowing for this increase in mammary gland differentiation. Progesterone is also needed for lobular alveolar growth in the mammary gland [128]. While colostrum yield is reduced in both restricted and overnourished ewes [65,124], when ewes are fed similarly throughout lactation, overfed ewes, but not restricted ewes, rebound in their milking ability to yield similar milk weights as CON ewes [65].

Not only does maternal diet impact the milking ability of the ewe, but the milking ability of her offspring and reproductive capacity of her grand-offspring [129]. This postnatal response may be linked to the anatomical differences observed in mammary gland development during fetal life. Fetuses from maintenance fed ewes had larger mammary gland weights at 100 days of fetal life compared to *ad libitum* fed ewes, without impacting mammary duct area or number or fetal weights [130]. Perhaps, this enhanced milk production is due to decreased fat pad hyperplasia and increased abundance of Mitogen-activated protein kinases (MAPK) and mTOR pathway signaling proteins within the fat pad [131].

### 4.1. Vascularity of the Mammary Gland

The mammary gland is a very dynamic organ that is influenced by gestational nutrition as evidenced by the alveolar proliferation and other cellular activity that may occur. However, it does not appear that maternal nutritional plane during gestation impacts the capillary vascularity of the mammary gland in sheep (Table 6) or beef cattle [122]. Interestingly, our laboratory has demonstrated that supranutritional levels of selenium fed during pregnancy increases mammary gland vascularity at birth, as well as by the end of a 20 day lactation (Table 6). Moreover, enhanced milk yields from ewes supplemented with supranutritional levels of selenium compared to adequately levels of selenium in the diet of ewes have been reported [65]. Investigations are on-going to determine what role enhanced vascularity may be contributing to increased milk yield. While selenium has been reported as an effective breast cancer reducing supplement [132], we hypothesize that the enhanced vascularity of ewe mammary glands resulting from supranutritional selenium supplementation may be advantageous from a milk production and offspring standpoint. While most breast cancers are associated with higher estrogen levels in women [133], we have not observed that supranutritional selenium supplementation alters estradiol-17β concentrations in circulation [86,127]. It is unknown, however, what concentrations of estradiol-17β are within the mammary gland itself.

**Table 6:** Impacts of maternal diet during pregnancy on vascularity and production of the mammary gland in ewes.

Treatment	Stage	Impact on Vascularity Compared to Control	Colostrum/Milk Production Compared to Control
**Nutritional plane**	**At birth**		
Control *vs.* nutrient restricted		NS *^, [125,126]^	Decreased 53%–70% ^[65,124]^.
Control *vs.* overnourished		NS *^, [125,126]^	Decreased 37%–58% ^[65,124]^.
	**Lactation Day 20**		
Control *vs.* nutrient restricted		NS *^, [126]^	Decreased 28% ^[65]^
Control *vs.* overnourished		NS *^, [126]^	NS ^[65]^
**Level of Selenium (Se) Supplementation**			
Control (0.3ppm) *vs.* Supra (3.0 ppm) Se	birth	Increased 20%–25% ^[125]^	NS *^, [124]^
Control (0.3ppm) *vs.* Supra (3.0 ppm) Se	birth	NS *^, [126]^	Increased 37% ^[65]^
Control (0.3ppm) *vs.* Supra (3.0 ppm) Se	Lactation Day 20	Increased 22% ^[126]^	Increased 10% ^[65]^

* NS = not significant at *p* > 0.05.

### 4.2. Maternal Small Intestine during Lactation

The shift from pregnancy to lactation typically includes an increase in voluntary feed intake in addition to the other metabolic and hormonal changes; therefore, the small intestine generally increases in mass during early lactation [52,134,135,136]. Increased intestinal mass during lactation could result from both increasing metabolic demand and intake. Research with murine models demonstrate the influence of metabolic demand of lactation on the small intestine. In lactating mice that had undergone intestinal resection, small intestinal mass increases 200%–300% to enhance nutrient acquisition, compared with a 60% increase in mass of controls [137]. Little is known about changes in vascularity of the small intestine during early lactation. In ewes, capillary area density decreased by 10% from Day 0–20 post-partum [52]. Because mass of the small intestine also increased by 40% during the first 20 day of lactation, this allowed for similar total vascularity between the two time periods. The decrease in capillary area density may result from a dilution of vascular area caused by the rapid increase in the mass of the small intestine. Furthermore, it appears that the metabolic pressure of lactation may rely less on the vascularity of the small intestine per unit area, and more on the increase in size of the small intestine to increase nutrient absorption capacity.

Few data demonstrate the influence of pre-partum nutrition on small intestinal measures during lactation. It is unknown if any of the changes that gestational nutrition causes on the maternal small intestine program its function long-term. Due to the plastic nature of the tissue, it appears that the small intestine changes rapidly during early lactation, especially in the face of adequate nutrition post-partum. In first parity ewes, we demonstrated that even when vascularity of the small intestine was affected by nutritional plane or supranutritional levels of selenium at parturition, the same measures were similar after being on common diets for 20 day of lactation [52]. In fact, when ewes were fed to meet their nutrient requirements for early lactation post-partum (*i.e.*, all treatments experienced an increase in nutrient intake), mass of the small intestine was similar for all ewes at Day 20 of lactation. Mass of the small intestine rebounds faster than ewe body weight, resulting in ewes that had previously been on a lower plane of nutrition having greater proportional small intestinal mass (g kg^−1^ body weight) than control ewes. This results from a greater increase in crypt cell proliferation in ewes that had been nutrient restricted compared with those that had been adequately fed during gestation [52].

To our knowledge, vascularity of the small intestine during lactation in response to previous plane of nutrition during gestation has only been reported in one study ([52]; Table 3). In this study, ewes that had been nutrient restricted during gestation have increased capillary surface density of the jejunum compared with control ewes at 20 days of lactation. Additionally, ewes previously overnourished during gestation had greater total vascularity compared with control ewes at 20 days of lactation. In both cases, these changes due to nutrition during pregnancy were consistent with those observed at parturition, suggesting that vascularity of the small intestine does not change as quickly as proliferation and mass. Despite this, previous intake of supranutritional selenium during gestation resulted in ewes having greater capillary size in the jejunum at 20 days of lactation when compared with control, even though these treatments had similar capillary size immediately post-partum. This supports the hypothesis that selenium suppresses vascular beds, as capillary size increased after return to an adequate selenium diet, although ewes had not likely yet returned to the selenium status of control. The role of VEGF and NO systems in altering vascular changes of the small intestine during early lactation is not clear. In this study, any differences of divergent treatments from control immediately post-partum subsided by Day 20 of lactation, including gene expression of angiogenic factors ([52]; Table 4).

Nutrient release by the portal-drained viscera (including small intestine) increases with milk production in ewes, suggesting that nutrient absorption by the small intestine increases with milk yield [138,139]. In our previous study, milk production for the first 20 days of lactation did move in a similar direction as vascularity of the small intestine for ewes that were nutrient restricted or fed supranutritional selenium during gestation [52,65]. (Table 3). We hypothesized that the small intestine impacts milk yield differences (decreased milk yield in ewes from lower planes of nutrition during gestation) by altering nutrient acquisition or by diverting nutrients from milk production to the rebuilding of body tissues including the gastrointestinal tract. Perhaps, during these times of high physiological demands, the intestine modulates its ability to secure more nutrients from luminal compared with arterial supplies. Data addressing this question are not available in the literature but would increase our understanding and potentially provide better positioning for management during physiological stress. Additionally, it is not known if small intestinal adaptation pre-partum is partially in preparation for lactation. Because the gastrointestinal tract responds rapidly to changes in nutrient intake, inappropriate nutrient intake pre-partum can alter the magnitude of its adaptation to lactation. Moreover, periparturient changes in the small intestine are necessary for adequate nutrient digestion and absorption to provide for milk yield, so this likely impacts lactation and nutrient availability for offspring during postnatal life.

## 5. Conclusions

The vascularity of the gastrointestinal tract, placenta, and mammary gland may be able to adapt to different nutritional stimuli, and in some cases, we know that nutrient exchange can be altered. It is imperative that we continue to investigate how the pregnant animal can not only survive under stressful nutritional paradigms (*i.e.*, inappropriate nutrient supply, conditional increased nutrient demand, specific nutrient imbalances, *etc.*), but also how those adaptations may be impacting her ability to deliver nutrients to the developing offspring. Continued effects to understand how vascularity and other factors associated with nutrient extraction may be key for enhancement of nutrient transfer efficiency.
